# Experimental Models to Study Epithelial-Mesenchymal Transition in Proliferative Vitreoretinopathy

**DOI:** 10.3390/ijms24054509

**Published:** 2023-02-24

**Authors:** Azine Datlibagi, Anna Zein-El-Din, Maxime Frohly, François Willermain, Christine Delporte, Elie Motulsky

**Affiliations:** 1 Laboratory of Pathophysiological and Nutritional Biochemistry, Université Libre de Bruxelles, 1070 Brussels, Belgium; 2 Department of Ophthalmology, Erasme Hospital, Hôpital Universitaire de Bruxelles, Université Libre de Bruxelles, 1070 Brussels, Belgium; 3 Department of Ophthalmology, CHU St Pierre and Brugmann, Université Libre de Bruxelles, 1000 Brussels, Belgium

**Keywords:** proliferative vitreoretinal diseases (PVDs), experimental models, epithelial-mesenchymal transition (EMT)

## Abstract

Proliferative vitreoretinal diseases (PVDs) encompass proliferative vitreoretinopathy (PVR), epiretinal membranes, and proliferative diabetic retinopathy. These vision-threatening diseases are characterized by the development of proliferative membranes above, within and/or below the retina following epithelial-mesenchymal transition (EMT) of the retinal pigment epithelium (RPE) and/or endothelial-mesenchymal transition of endothelial cells. As surgical peeling of PVD membranes remains the sole therapeutic option for patients, development of in vitro and in vivo models has become essential to better understand PVD pathogenesis and identify potential therapeutic targets. The in vitro models range from immortalized cell lines to human pluripotent stem-cell-derived RPE and primary cells subjected to various treatments to induce EMT and mimic PVD. In vivo PVR animal models using rabbit, mouse, rat, and swine have mainly been obtained through surgical means to mimic ocular trauma and retinal detachment, and through intravitreal injection of cells or enzymes to induce EMT and investigate cell proliferation and invasion. This review offers a comprehensive overview of the usefulness, advantages, and limitations of the current models available to investigate EMT in PVD.

## 1. Introduction

Proliferative vitreoretinal diseases (PVDs) are a vision-threatening group of pathologies that comprise proliferative vitreoretinopathy (PVR), epiretinal membranes (ERM), and proliferative diabetic retinopathy (PDR). Similarly, membranes found in neovascular age-related macular degeneration (nAMD) share common pathological pathways with PVD [1]. PVDs are characterized by avascular or fibrovascular membranes developing above, inside and/or beneath the retina. While PVR usually occurs after retinal detachment or ocular trauma, due to an excessive wound healing response, membranes in nAMD and PDR are triggered by local inflammation or oxidative stress and develop when the diseases are left unchecked or fail to respond to treatment [2,3,4,5,6,7]. Clinically, PVD membranes exert a vitreoretinal traction which may lead to retinal detachment and are responsible for most of secondary retinal detachments following initial surgical repair. The incidence of PVD is expected to rise in the coming decades due to the increase of diseases and risk factors responsible for PVD development [4,6,7,8,9,10,11,12]. However, surgical peeling of these membranes by specialized surgical teams remains the sole therapeutic option to this day, limiting patients’ access to treatment and burdening healthcare systems. Commonly, peeling of these membranes involves the use of dyes and drugs to stain the internal limiting membrane (ILM) on the neuroretina and/or vitreous to help visualizing these transparent structures and avoid iatrogenic lesions of the retina. However, compounds frequently used to stain the ILM and vitreous, such as Brilliant Blue G and triamcinolone acetonide, respectively, may diffuse through the ILM and exert a cytotoxic effect on the neuroretina and retinal pigment epithelium (RPE), potentially limiting patients’ post-operative visual prognosis [13,14].

**Figure 1:**
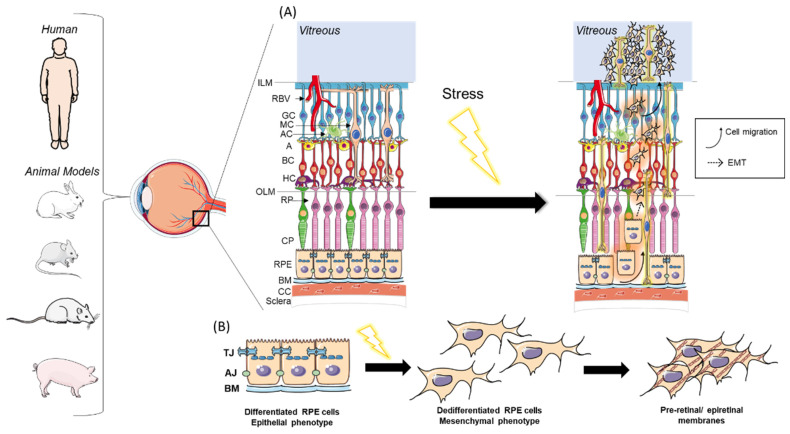
Role of EMT in PVR. (**A**) Depiction of PVR formation after stress factors’ impact on retinal cells: EMT and migration of RPE cells through retinal cellular layers (highlighted in red) as well as gliosis of Müller cells (highlighted in yellow). (**B**) EMT of RPE cells due to stress, leading to preretinal and epiretinal membranes formation: Following disruption of BRB or compromised retinal architecture due to stress (ageing, hypoxia, inflammation, or traumatism), normal cobblestone-shaped RPE cells lose their tight and adherent junctions, their apical basal polarity, and obtain a mesenchymal phenotype, which increases their migrative and proliferative abilities. These RPE cells undergoing EMT then migrate through the different retinal layers to form preretinal and epiretinal membranes. A: Amacrine cell; AC: Astrocyte; AJ: Adherens Junctions; BC: Bipolar cell; BM: Bruch’s Membrane; CC: Choroidal Capillaries; CP: Cone photoreceptors; EMT: Epithelial–Mesenchymal Transition; GC: Ganglion Cell; HC: Horizontal Cell; ILM: Inner Limiting Membrane; OLM: Outer Limiting Membrane; RBV: Retinal Blood Vessels; RP: Rod photoreceptor; RPE: Retinal Pigmented Epithelium; TJ: Tight Junctions. The Figure was partly generated using Servier Medical Art, provided by Servier, licensed under a Creative Commons Attribution 3.0 Unported license.

RPE and Müller cells have been identified as the main cell types involved in PVD. Commonly, following disruption of the blood retinal barrier caused by chronic pathologies, retinal tear, retinal detachment, or penetrating ocular trauma, RPE cells acquire myofibroblast characteristics, allowing them to migrate and form the contractile membranes found in PVD [3,5,15,16,17,18,19]. This process, named epithelial-to-mesenchymal transition (EMT), can occur in both physiological conditions such as embryogenesis and wound healing, and pathological conditions such as cancers and tissue fibrosis. EMT is characterized by a loss of apical-basal polarity, a switch in the expression of cytokeratins to vimentin, and increased cellular motility and invasive ability [18,19,20,21,22]. Similarly, endothelial cells can also undergo a process called endothelial–mesenchymal transition, as seen in embryogenesis, cardiac fibrosis, and fibrovascular membranes found in PDR and nAMD [5,15,16,20]. Müller cells also play a significant role in PVD development, mainly through the secretion of cytokines and growth factors, leading to gliosis and proliferation [23]. The detailed role of EMT in PVD has been summarized in Figure 1.

Despite sharing similar mechanisms during their development, members of the PVD spectrum have mainly been investigated separately. Therefore, it is of paramount importance to study EMT, a process occurring in all PVD, to better understand the pathogenesis of these diseases. Since proliferative membranes in PVR often develop within a few weeks following RPE layer disruption, experimental models mimicking acute or subacute development of membranes to study EMT as a key process of PVD will be useful to identify alternative and/or complementary treatments to improve patients’ visual prognostic [2]. A review published in 2017 sorted and described the animal models that have been used until now to study PVR and to perform pharmaceutical investigations [24]. However, the use of these in vivo models to study EMT in PVR has not been described. Furthermore, to the best of our knowledge, no review describing currently used in vitro PVR models has ever been published.

This review provides an overview of the currently existing in vitro and in vivo PVR models, as PVR membranes are mainly characterized by EMT of retinal cells. The review will also highlight the use of PVR models for research purposes as well as their advantages and limitations to study the EMT process involved in the pathogenesis of PVD.

## 2. In Vitro Models of PVR

Most in vitro models of PVR rely on immortalized cell lines, pluripotent stem cells, and primary cells, mainly RPE and Müller cells, which represent the most abundant cells found in PVR membranes. However, only a few published reports have attempted to use cells directly isolated from PVR membranes.

In vitro models provide several benefits over in vivo models such as the ease of access to cell lines, a lower cost, and the possibility to obtain highly reproducible models. However, in vitro models also possess limitations compared to in vivo models, and the most widely used cell lines often possess abnormal karyotypes, which may restrain the conclusions drawn from such models.

Research groups studying PVD in vitro have often had recourse to inducing EMT in cell lines or primary culture by exogenous adjunction of transforming growth factor β (TGF-β), tumor necrosis factor α (TNF-α) or other cytokines and growth factors. The proliferative and contractile properties of RPE cells after wound healing or in presence of animal vitreous have also been evaluated. Hereafter we describe the different in vitro models that have been used to this day to study PVR, with an emphasis on their advantages and drawbacks.

### 2.1. EMT Induction by Growth Factors or Cytokines

EMT induction by addition of TGF-β to the culture medium of RPE cells, first described in 2001, has become the most often used method to study EMT in PVR in vitro models [25]. Both TGF-β1 and TGF-β2, used in different cell lines and primary cells, seem to induce EMT mainly through the activation of the Smad signaling pathway [26,27,28]. The vast majority of studies treating RPE with TGF-β showed increased expression of EMT markers, modification of cell morphology towards a mesenchymal state as well as increased migration, proliferative and contractile abilities using wound healing, invasion, and collagen contraction assays [26,27,28,29,30]. Interestingly, a recent study showed that exosomes produced by TGF-β2-treated ARPE-19 cells induced EMT in normal ARPE-19 cells, which underlines the importance of the microenvironment to initiate EMT in RPE cells [31]. However, TGF-β alone does not suffice to induce EMT efficiently but requires a loss of cell–cell contact to initiate this process [32].

In 2010, a combination of TGF-β and TNF-α, added for the first time in RPE cell culture medium, revealed their synergistic effect to induce EMT [33]. Ever since, this combined treatment has only been used thrice on ARPE-19 and primary human RPE cells despite showing promising results to induce EMT in RPE cells, leading to the formation of membranes and fibrotic deposits [34,35,36].

Other growth factors and cytokines such as epidermal growth factor (EGF), TNF-α, interleukin 6 (IL-6), fibroblast growth factor 2 (FGF2), Gremlin or Factor Xa have also been used to induce EMT and study EMT markers, proliferation, migration, and morphology of RPE cells [29,30,37,38]. These models could provide alternatives to the TGF-β-induced EMT model but remain to be more extensively studied.

### 2.2. EMT Induction through Mechanical Stimulation

Few groups have studied the behavior of human induced pluripotent stem cell (hiPSC)-derived RPE cells, primary RPE cells, and immortalized human RPE cells in reaction to a wound healing assay mainly to investigate their proliferative ability and the effect of potential antiproliferative drugs. These models have also been used to investigate the cell contractile properties using collagen matrix contraction assays [27,39]. Surprisingly, it has been reported that exposition to normal vitreous fluid during wound healing tends to increase the fibrotic response of hiPSC-derived RPE cells [40].

Recently, two studies have revealed that low-density cell culture for an extended period potentiated EMT in TGF-β1-treated ARPE-19 cells and spontaneously induced EMT in human embryonic stem cell-derived RPE [30,41].

### 2.3. Advantages and Limitations of In Vitro PVR Models

To establish in vitro PVR models, research groups have mostly used human cell lines that have spontaneously developed from primary RPE and Müller cells (such as ARPE-19 and MIO-M1, respectively), as well as human, rabbit, mouse, rat, and porcine primary RPE cells. In the last decade, differentiated RPE cells derived from human pluripotent stem cells have also been used to explore the EMT process involved in PVD pathogenesis, as they share functional and mature characteristics of native human RPE cells [27,28,36,42,43,44].

**ARPE-19**, a spontaneously arising human RPE cell line, has been most widely used to investigate EMT in PVR and other retinal disorders. ARPE-19 have been successfully used in all the aforementioned in vitro PVR models and offer an easily accessible source of RPE cells [45,46]. However, the use of these cells does not come without any drawback, as they show an abnormal karyotype and a loss of key characteristics of differentiated RPE including the cobblestone morphology of RPE cells favoring a mesenchymal cell morphology, the apicobasal polarity and the expression of some RPE markers [47,48]. Therefore, ARPE-19 may have already undergone partial EMT and do not represent an ideal cell line to study the initiation of EMT in PVD models [48,49]. This limitation can however be overcome by proper differentiation of ARPE-19 into mature RPE cells through addition of pyruvate in the culture medium for three to four months [50]. Recently, a rapid differentiation protocol using culture medium supplemented with nicotinamide has been reported to allow the cells to form a polarized epithelium with cobblestone appearance but lacking pigmentation within two to four weeks and to regain RPE functions [51]. Therefore, the use of differentiated ARPE-19 may represent an affordable and easy-to-handle in vitro model to study EMT induction in mature RPE cells and to mimic PVD pathogenesis.

**Other spontaneously immortalized human Müller and RPE cell lines**, such as MIO-M1 and D407, respectively, have also been used to investigate PVR [42,52]. However, they have been seldom used to study the EMT process in PVD. Furthermore, the D407 cell line shows similar limitations to the ARPE-19, such as an abnormal karyotype and lack of differentiated RPE characteristics [49].

**Primary RPE cells** originating from human, rabbit, mouse, and swine have been isolated from ocular globes and used for in vitro PVR studies. As for the ARPE-19, EMT induction in primary cells has been achieved by treatment with EGF, TGF-β and/or TNF-α, by mechanical wound healing or by cultivating the cells in presence of vitreous fluid [27,35,53,54]. The use of primary cells allows researchers to establish RPE sheets possessing in situ RPE features before inducing EMT, without biases of potential abnormal karyotypes of cell lines. However, all research teams do not have access to human donors shortly after their death or to animal eyes, nor possess the expertise to perform RPE isolation, which limits studies relying on primary cells.

**Pluripotent stem-cell-derived RPE** represents an alternative to obtain fully mature cells exhibiting all characteristics of native RPE and may therefore be the ideal cell type to develop in vitro PVR models. Furthermore, hiPSC can be obtained from human with minimally invasive techniques, such as skin biopsy or blood sampling [27,41]. However, hiPSC culture and differentiation into RPE cells is costly and time-consuming, whether the differentiation is spontaneous or guided, therefore limiting its use in routine research [40,49].

**Cells isolated from human PVD membranes** have been maintained in culture or subjected to TNF-α treatment to explore EMT and their proliferative and contractile properties [55,56,57,58]. This model allows to investigate the characteristics of proliferating cells composing the pathological membranes but has been very scarcely used due to the limited access to human samples. Furthermore, this model cannot be considered a true in vitro PVD model since the cells are already in an end-stage EMT prior to being isolated, even though their invasive properties increased after exposition to TNF-α and/or TGF-β [56,57].

## 3. Animal Models

Several animal models have been developed to study EMT in PVD. Animals used for this purpose mainly consist of rabbits, mice, and rats. In vivo animal models are valuable for PVD evaluation as they can be used to mimic human disease by inducing post-traumatic PVR or other pathological PVD and investigate novel therapeutics. Nonetheless, animal PVDs’ pathophysiology and clinical expression can be different from the human disease which limits the extrapolation of animal studies on human PVR. Table 1 summarizes the characteristics of animal PVR models.

### 3.1. Rabbit PVR Models

Rabbits are some of the most common animals used for in vivo experimental PVR research. This species presents many advantages, such as its ease of acquisition and handling, a small lens size and a voluminous posterior chamber close to the human vitreous’ volume, which allows an easy access to the retina and better visualization of developing PVR [80].

PVR has been mainly induced in rabbits by intravitreal injections of cells or biologically active compounds and by ocular trauma through surgical means. These PVR models have been developed to study various components involved in PVR pathogenesis. Indeed, the proliferative and inflammatory vitreous reaction following the injection of cells, cytokines, growth factors and/or other blood components reproduce different stages of PVR [73,115,116]. In addition, surgical techniques mimicking ocular trauma reproduce the most frequent cause of PVR, disrupting the blood–retinal barrier (BRB), and allowing the subsequent activation and recruitment of macrophages, fibroblasts, and glial cells [37,117]. Interestingly, PVR has also been induced by associating ocular trauma with intravitreal injections or the simultaneous injection of different cell types and/or active compounds [52,70,117,118]. Such combination allows a concomitant representation of various aspects of PVR occurring at different stages, leading to better mimicking the human disease.

The first described injection model reproducing PVR in rabbits was described in 1984, based on the induction of a “fibroplasia” by injecting dermal connective tissue into the vitreous [119]. Since then, the intravitreal injection of cells or other compounds has been widely used in rabbit PVR models [37,59,61,70].

#### 3.1.1. Cell-Induced Rabbit PVR Models

**Homologous or heterologous intravitreal injections** of fibroblasts of various origins [59,60,120], human RPE cells [61,62,63], transfected ARPE-19 cells [64,121], primary RPE cultures [66], Müller cells [66], macrophages [67,121], or platelet-rich plasma (PRP) [122] have been used to develop PVR model. The most frequently studied model is the intravitreal injection of fibroblasts [59,60,120]. Such injections can trigger the development of epiretinal and intravitreal proliferative membranes within a few days after injection, due to the host reaction to these exogenous fibroblasts, ultimately leading to retinal detachment [115,123]. The injection of fibroblasts triggers an inflammatory infiltration, migration of RPE cells from the subretinal space, and loss of the initial RPE hexagonal shape towards a fibroblast-like appearance [124].

**PRP and blood derivatives’ injection** stimulates EMT, mainly through the secretion of growth factors and active mediators provided by platelets, such as platelet-derived growth factor (PDGF) and vascular endothelial growth factor (VEGF) [122,125]. Retinal blood vessels’ occlusion by platelets further stimulates neovascularization and proliferation [126]. Furthermore, co-injection of PRP and fibroblasts leads to the development of higher stage PVR with intense intraocular proliferation and preretinal vascularization [127,128].

#### 3.1.2. Biologically Induced Rabbit PVR Models

Several groups have also performed intravitreal injections of biologically active compounds such as dispase [76,129], TGF-β [70], VEGF [80,81], recombinant human IL-1β [87], xanthine and xanthine oxidase [82], to induce inflammatory and proliferative reactions and prompt PVR development. Dispase is an easily accessible enzyme that induces histological changes in the retina with very few side effects [76]. As a metalloprotease, dispase dissociates cells from their surrounding matrix, leading to RPE cells’ exposition and disrupting the vitreoretinal continuity [130]. Leakage and recruitment of endogenous cells such as fibroblasts, macrophages, and glial cells then follow, driving the expression of growth factors and cytokines which will stimulate the cells giving rise to PVR [77].

#### 3.1.3. Cell- and Biologically Induced Rabbit PVR Models

Some of the compounds and cells previously described have also been used in conjunction to induce PVR. For instance, some cells (RPE cells, human fibroblasts, heterologous fibroblasts etc.) were injected along with cytokines or other cells (PRP rich in trophic factors and cytokines) [52,118]. The co-injection of cytokines and PRP leads to dissociation and migration of RPE cells, mainly stimulating the proliferative process with moderate inflammation and subsequent development of PVR exhibiting thicker ERM compared to cytokine injection alone [118].

#### 3.1.4. Surgically Induced Rabbit PVR Models

Since ocular trauma is the main cause of PVR in humans, several research groups have attempted to mimic trauma through surgical techniques to induce PVR in rabbits. These models involve performing open-globe injury or vitrectomy followed by retinotomy or cryopexy [37,83,84]. Multiple features of human PVR can be represented in these models, such as retinal tear and BRB disruption that follow retinal detachments or traumatisms in human PVR. Epiretinal scarring as well as proliferation processes involving endogenous cells’ recruitment reproduce more accurately PVR pathogenesis compared to models where injections of exogenous cells and agents are performed [131]. Furthermore, PVR onset time in mechanical models is around 4–12 weeks, similarly to observations made in humans, which allows the evaluation of drugs and long-run interventions [132]. However, surgical/traumatic models might exacerbate proliferation by excessive vitreous hemorrhage related to the experience of the surgeon, which renders these models less reproducible.

#### 3.1.5. Cell- or Biologically Induced Rabbit PVR Models following Surgery

**Surgical techniques combined with injection** of one or several cell types and/or cytokine have been used to obtain a more reliable PVR model and to better investigate the physiopathology of human PVR where a retinal tear is often the main precursor of the disease [85,86,87]. Retinal defects, potentialized by the injected cytokines, allow the migration and proliferation of various cells into the vitreous through the retina or the interaction of injected cells with leaking cytokines, cells, and growth factors. These cells will then induce subsequent epiretinal membranes, surface wrinkling retinopathy and star-fold-like configurations 4 weeks after surgery [87]. Nonetheless, as rabbit retinas are less vascularized compared to human, these models are not ideal to study the actual impact of BRB disruption and subsequent ERM formation [65,88]. In fact, rabbit retinal vascularization pattern (merangiotic) is different compared to humans (euangiotic/holangiotic) with PVR in rabbits beginning on or around the medullary rays where retinal vasculature is present, the rest of the rabbit retina being avascular [133]. Retinal vasculature in humans plays an important role in PVR development as the anatomical disturbance of the retina and BRB disruption play a significant role in subsequent migration of inflammatory cells and proteins. Furthermore, newly formed retinal vessels following retinal detachment are common in PVR and may be sources of growth factors and inflammatory cells leading to ERM formation [134].

**Cell injection following gas vitrectomy**, using mainly perfluoropropane (C3F8), leads to PVR development within 7 to 28 days [128]. This technique allows emptying the vitreous chamber before cell injection, lowering the intraocular pressure, and softening the ocular globe easing intravitreal manipulations and subsequent injections rendering the model more reproducible [128]. This model is particularly interesting in rabbits due to the smaller lens size compared with the eyeball which allows vitreous manipulations to be performed without damage to the lens or retina [89]. Such a procedure allows the posterior detachment of vitreous and preretinal membranes development, the latter attributable to the break of retinal cell-to-cell contact and the disruption of the BRB, happening occasionally with posterior vitreous detachment, leading to RPE cells, collagen fibers, myofibroblasts, growth factors and cytokine leakage into the vitreous [61,90].

#### 3.1.6. Advantages and Limitations of Rabbit PVR Models

Briefly, rabbit injection models can be preferred to traumatic and surgical ones due to their ease of manipulation and less traumatic application avoiding non-naturally occurring side lesions [76]. More specifically, intravitreal injection of cells allows the study of the proliferative stage of PVR due to the reaction of local cells to the injected ones while the injection of blood derivatives and active inflammatory components mimic the inflammatory reaction that leads to EMT and tractional membrane development [80,115,116,118]. However, the injection of exogenous cells bearing foreign antigens as well as the rapid disease development must be taken in account before selecting this type of model [76]. Some proliferative retinal diseases such as macular PVR and post-traumatic PVR can have a rapid onset and be simulated by the injection models [135]. However, other proliferative retinal diseases linked to chronic pathologies such as proliferative diabetic retinopathy and exudative age-related macular degeneration can take years for proliferation to be clinically apparent and thus cannot be accurately reproduced by these models.

On the other hand, traumatic and surgical models offer the advantage to stimulate locally available cells without foreign agent injection and with an onset time close to clinical PVR [132]. Different manipulations can also be facilitated by the lowered intraocular pressure and softening of the ocular globe occurring after gas injection [128]. Nonetheless, surgical techniques performed by different manipulators may not be perfectly reproducible without proper surgical expertise, leading to variable degrees of cell liberation and local reaction [86,117]. Furthermore, such procedures hold a high risk of severe vitreous hemorrhage that does not reproduce the clinical situation [136].

The use of PVR models combining surgical and injection techniques represents an alternative to the use of a sole technique. These models offer the advantages to induce PVR in a timely manner closer to human disease onset with changes in vitreous consistent with clinical PVR pathogenesis. These phenomena are attributable to the surgical BRB disruption and its subsequent effect on cell leakage and local reactions, as well as the inflammatory and proliferative benefits of the injected compounds [87]. Nonetheless, the vascularization of rabbits’ retina differs from humans, making these results less extrapolatable to human PVR [88].

### 3.2. Mouse PVR Models

Mice represent the second most frequently used in vivo PVR models due to their ease of handling, accessibility, and similarities to human physiology and anatomy. Furthermore, they offer the possibility to induce the disease by various means such as injections, surgery or genetic modifications. Most mouse models of PVR are mainly derived from the C57BL/6 strain which has been used for over 50 years and allows the generation of reliable transgenic models [38,97,104,106,137,138]. Mouse PVR models using intravitreal injections range from active compounds’ injection such as dispase or coagulation factors to cell injections of ARPE-19, preceded or not by surgical discontinuation of the RPE layer [37,92,93,103]. Transgenic models however are capable of developing PVR spontaneously and allow the evaluation of cell proliferation and ERM formation [105,106].

#### 3.2.1. Biologically Induced Mouse PVR Models

Intravitreal injection of dispase has been mostly used to induce PVR development in mice. The low cost of dispase and high reproducibility of the model, makes it ideal for PVR pathophysiological investigations without involving specific immune response [96,98]. In addition, the severity of induced PVR can be tuned by adjusting the concentration of dispase [94]. PVR severity can also be increased by the simultaneous injection of a coagulation factor (FXa) [37]. Furthermore, the inflammatory response of RPE cells can be triggered in such model by the simultaneous intravitreal hemorrhage mixed with vitreous profibrotic factors [37,99]. The RPE, macrophages, and glial cells enter an inflammatory phase participating in the creation of a sub- and epi-retinal membrane, with only the subretinal membrane containing RPE cells [94,99].

#### 3.2.2. Cell-Induced Mouse PVR Models

To the best of our knowledge, PVR induction by intravitreal injection of cells in mice has only been performed in two studies using ARPE-19 cells [92,93]. Such PVR model was characterized by the formation of an ERM resembling those observed in patients with PVR [92]. Injection of large quantities of exogenous cells may lead to significant inflammation due to the host reaction, making this model less representative of human PVR.

#### 3.2.3. Surgically Induced Mouse PVR Models

Gentle retinal detachment using forceps or silicon rubber needle has been performed to induce PVR in mice [26,100,101,102]. Such methods induce EMT by detaching the retina without damaging the underlying RPE. PVR development mostly occurs on the Bruch’s membrane and not on the surface of the detached retina [26,102]. However, released vitreous cytokines and BRB rupture lead to immunological reaction, making the experimental conditions difficult to control [100].

#### 3.2.4. Cell-Induced Mouse PVR Models following Surgery

ARPE-19 cell injection following intravitreal gas injection has been recently performed to induce PVR development in mice. Interestingly, intravitreal gas injection leads to posterior vitreous detachment and increases the severity of subsequent PVR formation possibly by facilitating cell migration [103]. This model mimics key pathological aspects of human PVR without compromising retinal integrity and represents a valuable model as it allows therapeutic and pathophysiological studies. However, injection of human RPE cells may induce excessive inflammation related to the use of foreign cells, which may bias results’ interpretations [103].

#### 3.2.5. Transgenic Mouse PVR Models

Transgenic mice models have been generated to spontaneously induce the development of PVR [104,105,106]. The transgenic specific overexpression of Rho-PDGF A and B in photoreceptor cells results in vascular and glial cell proliferation [104]. Rho-PDGF A transgenic mice allow glial cell proliferation, formation of an ERM made of astrocytes and RPE cells, and superficial vascularization of the retina. Rho-PDGF B transgenic mice are more interesting for studying vascular proliferative retinopathies as they develop deep retinal vascularization with an epiretinal membrane containing glial cells, endothelial cells, and pericytes. Considering the involvement of these growth factors in the pathogenesis of PVR, specific aptamers have been developed and used in therapeutic trials to modulate PVR [105]. Mice showing mutated Laminin Subunit Alpha 1 (LAMA1) retain a fetal vitreous vascularization and a pre-retinal glial membrane, and the ERM present is very similar to PVR [106]. Overall, these transgenic mice models present severe proliferative aspects allowing further study of the pathogenesis of proliferative retinopathies.

#### 3.2.6. Advantages and Limitations of Mouse PVR Models 

Mice represent interesting and valuable models to study PVR due to their short reproductive cycle, and ease of handling and housing. However, compared to rabbits, murine eyes are anatomically smaller and possess a large lens and small vitreous volume, making them more technically challenging for injection, surgery, and observation [103,139]. Furthermore, mouse retina is not completely comparable to the human’s due to the absence of macula [140]. Nevertheless, their accessibility and ease of genetic modification make them a particularly interesting model for studying pathophysiological mechanisms involved in PVR [38,94,98,104,106].

As in rabbits, PVR can be induced in mice by intravitreal injection of cells or active compounds, or by inducing trauma, to mimic some of the key steps of PVR development in humans, such as proliferation and inflammation. These models have been mainly used to study EMT initiation occurring in PVR [37,38,39,94,95,96,97,98]. Transgenic mice models are particularly interesting to study the inflammatory stage of PVR as the pathophysiological hypotheses suspected of being involved can be isolated and studied separately [38,94,98]. Since the different modalities of PVR induction do not allow pharmacological study of ERM and neovascularization, some transgenic models that spontaneously develop PVR are valuable to study the aggressive proliferative aspects and therefore the late stages of PVR.

Despite technical challenges related to large lens and small vitreous volumes, intravitreal injections remain interesting models of PVR as they minimize operative complications compared to PVR model induced by surgery. Nonetheless, caution must be taken during cell injections as they may prone an excessive inflammation that does not reproduce human PVR.

### 3.3. Rat PVR Models

A wide variety of rat strains (such as Long Evans, Wistar, Sprague Dawley, Brown Norway) has been used to develop PVR models, which could lead to problems of reproducibility. These models mainly rely on intravitreal injection of cells or active compounds such as dispase or PRP. Although rats possess large globes and smaller lenses, they have not been as frequently used as mice and rabbits to study PVR pathogenesis [41,112,141,142].

#### 3.3.1. Cell-Induced Rat PVR Models

Cells such as RPE, macrophages, or a combination of RPE and PRP have been used to induce PVR in rats [107,108,109,110,111]. PVR induction by intravitreal injection of ARPE19 has also been shown to induce vitreoretinal fibrosis, similarly to rabbits [107]. During EMT induction, the key step of RPE migration and proliferation in the intravitreal space can be enhanced by PRP co-injection with either ARPE-19 or primary RPE cells from 7-day-old Long-Evans rats (RPE-J) [108,110]. These co-injection models constitute valuable in vivo PVR models as they adequately mimic human PVR through the involvement of RPE cells, glial cells, macrophages, and fibroblasts in the fibrocellular membranes [111].

#### 3.3.2. Biologically Induced Rat PVR Model

Intravitreal or subretinal injection of dispase has been performed in rats to induce PVR [41,112]. Rat PVR models using dispase injection leads to disruption of retinal integrity and to EMT initiation. These models show similar benefits to mice models as they also allow investigation of EMT initiation and the testing of different therapeutic agents [41,112].

#### 3.3.3. Advantages and Limitations of Rat PVR Models

Compared to mice, rats possess larger ocular globes with proportionally smaller lens, a larger vitreous volume and present very similar advantages and disadvantages. However, the vast heterogeneity of rat strains used to investigate PVR in vivo limits the reproducibility of these models. Furthermore, rats have not been as extensively used to investigate PVR pathogenesis when compared to rabbits and mice [41,112,141,142].

### 3.4. Swine Models

Pigs have not been widely used to investigate the pathophysiology of PVR due to their heavy housing and maintenance cost, although pigs share several physiological and anatomical similarities with humans. Indeed, pigs possess a retinal structure characterized with a high density of photoreceptors, a holangiotic vascularization and a vitreous composition similar to humans. Moreover, due to the resemblance to human’s anatomy, the results obtained are the closest to human pathophysiology, making the pigs the best alternative to non-human primates for PVR studies. Furthermore, the use of pigs to develop PVR models has been motivated by the failure to translate all results found in other models to humans [113]. PVR induction in the porcine models is commonly performed through retinal detachment induced by subretinal balanced salt solution injection during vitrectomy, followed by an intravitreal injection of RPE associated or not with PRP [54,113,114]. PVR development in pigs is similar to humans, starting with the development of fibrotic membranes followed by tractional RD at day 14 [113,114]. Recently, a PVR model was obtained in minipigs by scraping the endogenous RPE layer following vitrectomy with induction of bleb retinal detachment [113].

## 4. Conclusions

This review provides a summary of existing in vitro and in vivo PVR models that allow investigation of the EMT process occurring in PVD, along with their advantages and limitations. Most in vitro models rely on the use of immortalized cell lines such as ARPE-19 cells due to their ease of access and lower cost compared to primary cells and hiPSCs. However, ARPE-19 cells lack several key features of mature RPE, which may limit correlation of experimental results to human PVD. A recent simple protocol allowing to rapidly differentiate ARPE-19 into mature RPE cells using nicotinamide would be very useful to study EMT initiation in PVD pathogenesis. Other in vitro models using primary and human pluripotent stem-cell-derived RPE cells offer the benefits of possessing most characteristics and features of native RPE cells, making them the best in vitro models for experimental investigation. However, the limited access to primary human RPE cells as well as the heavy cost and required expertise for primary RPE cells’ isolation and hiPSCs’ differentiation greatly limit their use in many research laboratories. Therefore, nicotinamide-induced differentiated ARPE-19 cells subjected to EMT through adjunction of TGF-β with or without TNF-α in the culture medium represents an attractive and relevant model to study PVR and PVD pathogenesis.

Among in vivo PVR models, rabbits and mice have been widely used to mimic PVR pathogenesis, mainly through intravitreal injection of dispase, fibroblasts, or RPE cells, or through surgical means mimicking penetrating ocular trauma. Mice and rabbits represent accessible animals to investigate PVD pathogenesis and therapeutic agents in preclinical models. Furthermore, mice offer the possibility of performing genetic modifications to study specific pathways that may be involved in PVD and identify potential therapeutic targets. Despite sharing many similarities with human retinal physiology and anatomy, making them the ideal in vivo model for PVR pathogenesis investigation, pigs have been rarely used, mainly due to their heavy housing and maintenance costs. Minipigs may represent an alternative to classical swine research as they need smaller facilities and offer similar benefits. However, the use of minipigs to study PVR needs further validation, as this model has so far only been used once.

Overall, both in vitro and in vivo PVR models present advantages and limitations. In vitro models provide a controlled environment for analyzing specific cellular and molecular processes involved in PVR pathogenesis. In vivo animal PVR models offer a more realistic representation of the disease but are limited by the difficulty in controlling variables and extrapolating findings to humans. These models represent complementary valuable tools to deepen our current understanding of PVD and to develop effective treatments for patients. Furthermore, PVR induction in 3D models such as organs-on-a-chip or 3D bioprinted outer retina may also provide innovating and interesting alternatives to study molecular mechanisms of PVD.

## Figures and Tables

**Table 1:** Characteristics of rabbit, mouse, rat and swine PVR models.

Model Type	Methodology	Strengths	Limitations	Therapeutic Investigations	Pathogenesis Studies
***Rabbit Models***					
***Cell-induced models***	*Intravitreal injection of:* 50,000–200,000 dermal, corneal or conjunctival fibroblasts [59,60] 250,000 cultured human RPE cells [61,62,63] 250,000 ARPE-19 cells [64] 200,000 primary homologous RPE cells [65] 50,000 Müller cells [66] 70,000–800,000 Macrophages [67]	Avoid major surgical side effects and anterior chamber lesions Evaluate proliferation in PVR Rapid onset models (3–4-day onset, ERM around day 28)	Not suitable for study of chronic PVR Injected exogenous cells induce inflammatory host reaction Absence of the traumatic component of PVR Absence of blood and plasma components’ activation	[59,64,65,68,69,70,71]	[66,72]
	Intravitreal injection of PRP (107 platelets in a volume of 30μL) or autologous blood [73,74,75]	Stimulate growth factors and cytokine secretion by platelets Induce proliferation Well-established, efficient, and cost-effective models Mimic high-risk human PVR	Absence of retinal detachment if not associated with traumatic lesions (PRP) Rapid PVR onset (2 weeks)	[74]	[75]
***Biologically induced models***	Intravitreal or subretinal injection of 0.05–0.07 UI dispase [76,77]	Recruitment of endogenous cells Eased access to dispase Histological changes close to human PVR Avoid surgical side effects and anterior chamber lesions Induction of high stages PVR	Inconsistency of study results due to uncertain purity of dispase solutions Prolonged exposure to dispase may induce cataract or lens subluxation Absence of the traumatic component of PVR	[78]	[79]
	Intravitreal injection of 10–20 μg of VEGF [80,81]	Mimics the neovascular proliferative aspect of PVR after 7 days	Rabbit retinal vascularization pattern different from humans	[80]	
	Injection of solutions containing 40 nmol Xanthine and 0.001 UI Xanthine oxidase [82]	Representative of the inflammatory PVR aspect ERM and retinal detachment 28 days after injection	Inflammatory host reaction in anterior and posterior chambers	[82]	
***Surgically induced models***	Unilateral surgical vitrectomy, retinotomy or cryopexy [83,84]	Representative of traumatic PVR aspect Disease onset 4 weeks post-surgery Stimulation of inflammatory response with cryotherapy	Variable extent of different surgeries Risk of being non-reproducible Risk of hemorrhage and excessive exudation of active components	[83]	[84]
	Open or closed-globe injury by scleral incision and fluid percussion injury device (FPI) on the center of the cornea at a 65° angle [37]	PVR developed in 2 weeks–6 months Mimics post-traumatic human PVR	Operator-dependent procedure Risk of anterior segment injury in closed-globe injury model		[37]
***Association of cell- and*** ***biologically induced models***	*Intravitreal injection of*: 106 ARPE-19 cells treated with 10 ng/mL TGF-β2 [70] 250,000 RPE cells and PRP [52]	Intense cellular proliferation and preretinal neovascularization Induction of high stages PVR	Rapid induction of high grade PVR (1–2 days)	[52,70]	
***Association of cell-,*** ***biologically and*** ***Surgically induced models***	Unilateral surgical intervention followed by fibroblast [85], PRP [86] or cytokine injections [87,88]	Representative of traumatic and inflammatory/proliferative PVR aspects PVR onset closer to human disease Evaluation of acute PVR phases	Rabbit retinal vascularization pattern different to humans	[85,86,88]	
	Gas compression by intravitreal injection of C3F8 or SF6 gas followed by cell injection (RPE, PRP and/or fibroblasts) [89,90,91]	Eased application due to small lens size Eased vitreous manipulations without damage to the lens or retina Liquification of vitreous avoiding anterior segment lesions Posterior detachment of vitreous and preretinal membrane development Emptying of vitreous chamber before other injections and subsequent lowering of IOP	Rapid onset as soon as day 3	[61,91]	
***Mouse Models***					
***Cell-induced models***	Injection of 50,000–160,000 ARPE-19 cells [92,93]	Injection of RPE cells involved in retinal remodeling Minimal operative complications	Injection of exogenous cells which may induce inflammatory host reaction	[92]	
***Biologically*** ***induced models***	Intravitreal injection of 0.1 U/µL–0.4 U/µL dispase [37,39,94,95,96,97,98,99] Injection of dispase/collagenase solution (0.02–0.4 UI/µL) [38,99]	Recruitment of endogenous RPE cells Eased access to dispase Reproducible technique Minimal operative complications	Prolonged exposure to dispase may induce cataract or lens subluxation Risk of intravitreal hemorrhage	[38,94,98]	[37,39,95,97,99]
***Surgically induced models***	Surgical retinal detachment with forceps without damaging the RPE layer [100,101,102] Lesion of peripheral retina using silicone rubber needle [26] Intravitreal injection of 0.5 µL of 100% SF6 gas followed by injection of 10,000 RPE cells [103]	Representative of traumatic PVR aspect Mimic key steps of human PVR	Operator-dependent model which limits reproducibility EMT occurs on the Bruch’s membrane	[26,100,101,102,103]	
***Transgenic models***	Homozygous rho/PDGF-B mice [104,105] Lama1 deletion [106]	Spontaneous development of proliferative membranes Investigation of neovascular and proliferative processes of PVD	Costly Limited accessibility to transgenic mouse species	[105]	
***Rat Models***					
***Cell-induced*** ***models***	Intravitreal injection of 106 ARPE-19 cells transfected with TP53BP2-specific siRNA [107] Intravitreal injection of 2.4 × 106 RPE-J cells and/or PRP containing 2 × 1010 platelets [108,109] Intravitreal injection of PRP containing 2.4 × 106 ARPE-19 cells [110] Intravitreal injection of 250,000 macrophages [111]	Minimal operative complications Induction of high-stage PVR Involvement of several cell types (RPE cells, glial cells, macrophages, and fibroblasts) as in human PVR	Injection of exogenous cells Risk of intravitreal hemorrhage	[108,109,110]	[108,109,110,111]
***Biologically induced models***	Intravitreal injection of 0,03 IU/µL of dispase [112] Subretinal injection of 3 µg of dispase [41]	Recruitment of endogenous RPE cells Stable and reproducible model	Prolonged exposure to dispase may induce cataract or lens subluxation Risk of intravitreal hemorrhage	[41,112]	
***Swine Models***					
***Association of cell- and surgically induced models***	Surgical retinal detachment induced by subretinal BSS injection followed by intravitreal injection of 8.108 RPE cells with or without PRP [54,113,114]	Best alternative to non-human primates Tractional retinal detachment 2 weeks after surgery and injection	Operator-dependent model which limits reproducibility Costly	[54]	[114]

ARPE-19: Spontaneously arising RPE cell line; C3F8: Perfluoropropane; CTGF: Connective Tissue Growth Factor; EMT: Epithelial-Mesenchymal Transition; ERM: Epiretinal Membrane; PRP: Platelet-Rich-Plasma; PVR: Proliferative Vitreoretinopathy; RPE cells: Retinal Pigment Epithelial cells; SF6: Sulfur hexafluoride; IOP: Intraocular Pressure; TGF: Transforming Growth Factor; VEGF: Vascular Endothelial Growth Factor; siRNA: silencing ribonucleic acid; TP53BP2: Tumor Suppressor P53-Binding Protein 2.

## Data Availability

Not applicable.
